# Heat shock protein 90 localizes to the surface and augments virulence factors of *Cryptococcus neoformans*

**DOI:** 10.1371/journal.pntd.0005836

**Published:** 2017-08-04

**Authors:** Sharanya Chatterjee, Utpal Tatu

**Affiliations:** Department of Biochemistry, Indian Institute of Science, Bangalore, India; University of Tennessee, UNITED STATES

## Abstract

**Background:**

Thermotolerance is an essential attribute for pathogenesis of *Cryptococcus* as exemplified by the fact that only two species in the genus, which can grow at 37°C, are human pathogens. Species which have other virulence factors including capsule formation and melanisation, but lack the ability to propagate at 37°C are not pathogenic. In another related fungal pathogen, *Candida albicans*, heat shock protein 90 has been implicated to be a central player in commanding pathogenicity by governing yeast to hyphal transition and drug resistance. Exploring Hsp90 biology in *Cryptococcus* in context of thermotolerance may thus highlight important regulatory principles of virulence and open new therapeutic avenues.

**Methodology/Principal findings:**

Hsp90 is involved in regulating thermotolerance in *Cryptococcus* as indicated by growth hypersensitivity at 37°C upon mild compromise of Hsp90 function relative to 25°C. Biochemical studies revealed a more potent inhibition of ATPase activity by pharmacological inhibitor 17-AAG at 37°C as compared to 25°C. Catalytic efficiency of the protein at 37°C was found to be 6.39×10^−5^μM^-1^. Furthermore, indirect immunofluorescence analysis using a specific antibody revealed cell surface localization of Hsp90 via ER Golgi classical secretory pathway. Hsp90 was found to be induced under capsule inducing conditions and Hsp90 inhibition led to decrease in capsular volume. Finally compromising Hsp90 function improved anidulafungin tolerance in *Cryptococcus*.

**Conclusions/Significance:**

Our findings highlight that Hsp90 regulates pathogenicity of the fungus by myriad ways. Firstly, it is involved in mediating thermotolerance which implies targeting Hsp90 can abrogate thermotolerance and hence growth of the fungus. Secondly, this study provides the first report of biochemical properties of Hsp90 of a pathogenic fungus. Finally, since Hsp90 is localised at the cell wall, targeting cell surface Hsp90 can represent a novel strategy to combat this lethal infection.

## Author summary

Thermotolerance is a pre-requisite for microbes to propagate successfully as human pathogens. In this study, we have investigated the role of Heat shock protein 90 in the pathogenesis and thermotolerance of *C*. *neoformans*, an environmental fungus that causes meningoencephalitis in humans. We show that thermotolerance of *Cryptococcus* critically depends on Hsp90 function as modest inhibition of Hsp90 function, robustly compromised growth of the fungus at 37°C with little effect at 25°C. This observation correlated with the fact that pharmacological inhibitor, 17-AAG also showed a more potent inhibition of ATPase activity of the protein at 37°C as indicated by a lower IC_50_ as compared to 25°C. Indirect immunofluorescence analysis using an antibody specific to CnHsp90 revealed cell surface localization of Hsp90. BFA sensitivity of such surface localization indicated involvement of ER-Golgi classical secretory pathway for this localization. Furthermore, inhibition of Hsp90 function not only abrogated the natural resistance of *C*. *neoformans* to cell wall targeting inhibitors echinocandins but also led to decrease in capsular assembly which is one of the classical virulence determinants of the pathogen. In all, this study provides the first detailed biochemical as well as functional insights into the role of Hsp90 in governing thermotolerance and augmenting virulence factors in *C*. *neoformans*.

## Introduction

All living cells are endowed with a heat shock response machinery which plays a protective role against stress. This machinery is inducible under heat shock and it provides cells the capacity to effectively withstand sub lethal temperatures and tolerance to many other stresses. This phenomenon has been shown to be exploited by pathogens wherein quick adaptation to divergent host environment is essential to establish a successful infection. Various classes of heat shock proteins such as Hsp60, Hsp70 and Hsp90 have been implicated to be involved in propagation of parasitic virulence. For instance, Hsp90 acts as a thermosensor in the malaria parasite wherein temperature stress is perceived as a cue for transition from one development stage to another [1]. In *Entamoeba* and *Giardia*, Hsp90 regulates the process of encystation [2,3].

Numerous observations have linked heat shock response with the pathogenic potential of fungi [4]. Hsp90, an essential molecular chaperone [1,3,5,6], has been shown to govern various aspects of *C*. *albicans* pathogenicity determinants including morphological yeast to hyphal transition [7], emergence of drug resistance [8–10] and biofilm formation [11]. Using genetic screens, it was shown that Hsp90 regulates thermal adaptation in *C*. *albicans* by downregulating Hsf1 [12]. Interestingly, in *C*. *albicans*, a 47kDa C terminal fragment of Hsp90 was shown to be present on the cell wall [13,14] and antibodies targeting the exposed Hsp90 were shown to inhibit infection [15]. In *Aspergillus fumigatus*, the role of Hsp90 in drug resistance [16], conidiation and maintenance of cell wall integrity has been well established [17]. In *Cryptococcus*, Hsp90 has been recently shown to be crucial for growth of the fungi and Hsp90 inhibitor radicicol has synergistic action with azoles [18].

The fungal kingdom comprises over 1.5 million known species present ubiquitously in the environment, most of which are either free living saprobes or commensals. In contrast to the large number of protozoa and viruses capable of infecting humans, there are only a few fungi which are human pathogens. Common leading systemic fungal pathogens include *Candida*, *Pneumocystis*, *Histoplasma*, *Aspergillus*, *Cryptococcus*, *Mucor*, *Rhizopus* and *Coccidioidomyces [19,20].* This is intriguing owing to the enormous diversity and the ubiquitous nature of the fungal kingdom. It is also noteworthy to highlight that most of the fungal infections are either restricted to the immunocompromised state of the host or may result from an accidental breach of the anatomical barriers. Furthermore, the advent of antibiotic therapies in the 1960s have also paved the way for fungal infections. Nonetheless upon summarizing the lessons learned from pathogenic and non-pathogenic fungi, we arrive at the conclusion that high body temperature in mammals and birds provide an innate physical barrier to the vast inoculum of ubiquitous fungal spores. Therefore, the ability to successfully survive at the physiological temperature of 37°C seems to be the utmost requirement in order to be pathogenic to humans.

The best example which underpins the importance of thermotolerance for virulence is seen in the genus *Cryptococcus* which comprises over 37 species, most of which are environmental and non-pathogenic to humans. Only two are human pathogens by virtue of their abilities to grow at 37°C implicating the importance of thermotolerance for pathogenesis [21,22]. Non-pathogenic species of *Cryptococcus* such as *C*. *podzolicus* are equipped with the other critical pathogenicity armours including capsule formation and melanisation, however they lack the ability to propagate at 37°C [23], indicating thermotolerance is the fundamental requirement for pathogenicity. However, the mechanism of growth at elevated temperature with respect to heat shock response has long been enigmatic.

In this study, we have tried to investigate the role of Hsp90 in thermotolerance of the fungus *C*. *neoformans*. We find that *C*. *neoformans* critically depends on Hsp90 machinery for survival at 37°C as indicated by hypersensitivity to Hsp90 inhibition at 37°C (human body temperature) as compared to 25°C (environmental temperature). Also, we have investigated Hsp90 mediated thermotolerance at the biochemical level by characterization of ATPase activity and its inhibition by pharmacological inhibitor. Hsp90 was found to be upregulated under capsule inducing conditions, and immunofluorescence analysis showed that Hsp90 is localized on the fungal cell surface. We also find that Hsp90 governs critical aspects of capsule regulation including capsule formation and maintenance around the cell wall. Furthermore, Hsp90 inhibition compromises intrinsic resistance of *Cryptococcus* to echinocandins–the only class of antifungals which targets the fungal cell wall. In all, our study establishes the involvement of Hsp90 in thermotolerance, cell wall integrity and capsulation processes in *C*. *neoformans* which are the most essential virulence determinants of the pathogenic fungus.

## Methods

### Strains and culture conditions

*C*. *neoformans* strain MTCC 1353 and clinical isolate was a kind gift from Dr. R Ravikumar, NIMHANS, Bangalore, India. All isolates were maintained at −80°C in 25% glycerol. Isolates were grown in either YPD (1% yeast extract, 2% bactopeptone, 2% glucose) or Sabouraud Dextrose broth unless otherwise stated. 2% agar was added for solid media.

### Minimum inhibitory concentration assays

Susceptibility to drugs was determined in flat bottom, 96-well microtiter plates using broth microdilution protocol. Minimum inhibitory concentration (MIC) tests were set up in a total volume of 0.2 ml/well with 2-fold dilutions of radicicol (RAD) and anidulafungin (AF). RAD gradients were typically from 5nM to 50μM with the following concentration steps in nM: 5, 50, 500, 1000, 5000, 10000, 20000, 30000 and 50000. AF gradients were used in the following concentration steps in μg/ml were: 32, 16, 8, 4, 2, 1, 0.5, 0.25. Cell densities of overnight cultures were determined by haemocytometer and dilutions were prepared such that ∼10^3^ cells were inoculated into each well. Plates were inoculated at the indicated temperatures. MIC_50_ was defined as the concentration of drug reducing growth by 50% relative to the wells containing no drug. Dimethyl sulfoxide (DMSO) was the vehicle control for radicicol (RAD) and Anidulafungin (AF). Absorbance was determined spectrophotometrically at 600 nm and was corrected for background from the corresponding medium. All drugs were purchased from Sigma Aldrich. To determine nature of interaction between Hsp90 inhibitor RAD and AF, we calculated the fractional inhibitory concentration by the following formula: ∑FICs = (MIC_50_ of AF in combination/ MIC_50_ of AF alone) + (MIC_50_ of RAD in combination/ MIC_50_ of RAD alone). ∑FIC values <0.5 indicates substantial synergism and 0.5–1.25 indicates an additive interaction.

### Spotting assays

Five-fold dilutions of cells (from ∼1×10^6^ cells/ml) were spotted onto SD agar media after growth assays as indicated and incubated at the indicated temperatures. Plates were photographed after 2–3 days.

### Immune blot analysis

*C*. *neoformans* cells grown till mid log phase were collected by centrifugation and the pellet was washed three times with ice cold PBS. The cells were lysed using ice cold lysis buffer containing 50 mM Tris (pH 7.4), 1% Triton X 100 containing 1 mM EDTA with an equal volume of 1-mm-diameter glass beads by multiple rounds of vortexing (BioSpecProducts, Inc., Bartlesville, OK). After disruption, the samples were centrifuged at 20,000 g for 15 min at 4°C to remove the glass beads, unbroken cells, and particulate debris from the homogenate. After centrifugation, the soluble protein fraction was collected, the protein content was measured by the Bradford assay. The proteins were stored at—80.0°C for further analysis.

Protein samples were mixed with one-fifth volume of 6× Laemelii buffer, boiled for 5 minutes, and resolved on a 10% SDS-PAGE gel. Protein was blotted onto PVDF membrane and blocked with 5% skim milk in tris buffered saline with 0.1% tween. Antibodies were used in the following dilutions: Anti CnHsp90 antibody (1:5000), Anti PGK antibody (1:5000), Anti tubulin antibody (1:2000).

Exponential phase cells were harvested and washed thrice with sterile PBS and resuspended in 10 mM phosphate buffer (PBS) (pH 7.4 containing 1% (v/v), βME at 25°C and 37°C. After 1h treatment, the cells were spun and the supernatant fluid was concentrated and suspended in Laemelli buffer followed by immunoblot analysis as described above.

### Cloning and purification of recombinant CnHsp90

Total RNA was isolated from yeast culture using the standard Trizol method and cDNA was prepared by RT PCR. Cn Hsp90 was amplified from *C*. *neoformans* cDNA using the following primers: 5’ CCCCCGGATCCATGTCCACCGAGACCTTTGG (forward primer) and 5’ CCCCCCGAATTCTTAGTCAACCTCCTCCATGGAG (reverse primer). Amplified product of 2157 bp was cloned in pRSET-A vector as a 6x-His tag fusion protein and transformed into competent *E*.*coli* DH5α cells. Confirmation of positive clones was done upon insert release by restriction digestion. For purification, His-tagged Cn Hsp90 was expressed in *E*. *coli* p LysS strain. Cells were grown at 37 °C till the optical density at 600 nm reached 0.6. Induction was done with 0.5mM IPTG at 16 °C. Ni-NTA column was used to purify CnHsp90 to homogeneity.

### K_d_ determination for ATP and 17-AAG binding using fluorescence spectroscopy

Fluorescence measurements were carried out in a Perkin Elmer fluorescence spectrophotometer as reported previously [6]. 20 μg of CnHsp90 was incubated with varying concentrations of ATP (100μM-3000μM) in binding buffer (40 mM HEPES-KOH buffer pH 7.4, 5 mM MgCl_2_ and 100 mM KCl) and tryptophan fluorescence was measured by scanning the emission spectrum in the wavelength range of 300–400 nm at excitation at 280 nm. The intensity at λmax 340 nm was selected for calculations. Difference in intrinsic fluorescence of protein alone and in presence of ligand was plotted against ligand concentration. Binding curve was analyzed using GraphPad Prism 5.0 software using non-linear regression analysis with single site-specific binding. Similarly, determination of 17-AAG binding was performed by incubating 20 μg protein in binding buffer (50 mM Tris and 1 mM EDTA) with varying concentrations of 17-AAG (500 nM– 70 μM). The final concentration of DMSO in the assay was 1%.

### ATPase assay

1.5 μM of CnHsp90 was incubated with varying concentrations of ATP (50 to 4000 μM) as previously described [24].The assay buffer contained 40 mM Tris Cl buffer, pH 7.4, 100 mM KCl, 5 mM MgCl2. [γ32P] ATP of specific activity of 0.55 Ci/mmole was used as a tracer. The reaction mixture was incubated at 25°C and 37°C for 1 hour. Thin layer chromatography was performed on polyethyleneimine-cellulose sheets (Merck) wherein the mobile phase contained 0.5M LiCl, 0.5mM EDTA and 2N formic acid. To rule out nonspecific ATPase activity due to copurifying proteins, 300 μM of Hsp90 inhibitor 17-AAG was used as control. The TLC sheets were dried and analyzed by phosphor imaging. The spots corresponding to phosphate and ATP were quantitated using Image Quant software (Fujifilm). Final Hsp90 ATPase activity was calculated by subtracting the activity in the presence of 17-AAG from the total ATPase activity. Data was analyzed using GraphPad Prism 5.0 software using Michaelis-Menten kinetics.

For ATPase inhibition assay, purified CnHsp90 was incubated with a saturating concentration of ATP (2 mM) and 17-AAG concentration was varied from 2.5 μM to 150 μM. 300 μM 17-AAG was used in control reaction. Percentage residual ATPase activity was plotted against log of concentration of inhibitor and the result was analyzed using GraphPad Prism 5.0 as described above.

### ELISA

ELISA was performed with intact yeast cells to confirm surface association of Hsp90 in *C*. *neoformans* as described previously [25]. Briefly, 5×10^5^ live yeast cells/well were incubated in 96-well polystyrene plates (Costar 9018; Corning Inc., New York, USA) for 2 h at 25°C and 37°C in PBS. Unattached yeast cells were removed by washing with PBS. Blocking was done for 1 h using 2% BSA (Sigma–Aldrich) in PBS supplemented with 0.05% Tween-20. The plates were washed three times with 0.1% Tween-20 in PBS and incubated with anti-CnHsp90 antibody that was serially diluted as indicated in the blocking solution. After 1 h incubation, the plates were washed and incubated with peroxidase-labeled anti-rabbit IgG (Sigma–Aldrich) and incubated for 1 h. Serologic reactions were measured by the addition of TMB (Invitrogen) and determined by OD measurements at 450 nm. Polyclonal antiserum against PGK raised in rabbit was used as a negative control. No antibody and no antigen controls were also used as negative controls.

### Indirect immunofluorescence analysis

Yeast cells were grown to log phase and washed with PBS. Vesicle trafficking inhibitors included BFA and NEM at concentrations previously used in similar studies [26]. A single colony on solid YPD medium was cultured overnight in liquid YPD medium. Cells were washed with PBS and 10^6^ cells were added into 3 ml of liquid YPD containing vesicle trafficking inhibitors BFA (25μg/ml) and NEM (500μM) and further cultured in a shaking incubator at 25 °C and 37°C. Cells were then fixed with paraformaldehyde for 30 mins. Blocking was done with 3% BSA in phosphate-buffered saline for 1 h. Primary antibodies against CnHsp90 was diluted in phosphate-buffered saline and incubated for 2 h. Following three washes with PBS, sample was incubated with fluorescein isothiocyanate-conjugated anti-rabbit secondary antibody. Three washes were given to the coverslips maintaining gentle agitation. The coverslips were mounted on glass slides with 90% glycerol containing 2% DABCO and Calcaflour white and visualized under a confocal laser scanning microscope (Leica TCS SP8). For cell surface association studies, it should be noted that permeabilization step was not carried out.

### Capsule formation

A single colony from solid YPD medium for each strain was cultured overnight at 37 °C in liquid YPD medium. For capsule induction, 10^6^ cells were added into 3 ml of capsule inducing medium (10% fetal bovine serum in PBS) and further incubated at 37 °C for 12 hours with shaking. Capsular staining was done by India ink and examined by Zeiss microscope. Hsp90 inhibitors RAD (0.5μM) was added either initially in the capsule inducing medium or after 12 hours of induction. Cell viability was examined by Trypan blue dye exclusion method to rule out cell death due to RAD treatment. To calculate the capsule volume, the diameters of the whole cell (*D*_w_) and the cell body (*D*_c_) were measured with Zeiss Zenlite software. Capsule volume was defined as the difference between the volume of the whole cell (yeast cell plus capsule) and the volume of the cell body (no capsule) as previously described [27]. Following equation was used: volume of a sphere as 4/3 × π × (*D*/2)^3^. 40 cells were measured for each experiment on at least three separate occasions.

### Statistical analysis

All results were reported as Mean ± S.E.M. Grouped data was statistically analyzed using one-way ANOVA. Two-tailed P-test was used for paired comparisons. All analysis was done using GraphPad Prism 5.0.

## Results

### Hsp90 is critical for thermotolerance of *C*. *neoformans*

**Fig 1:**
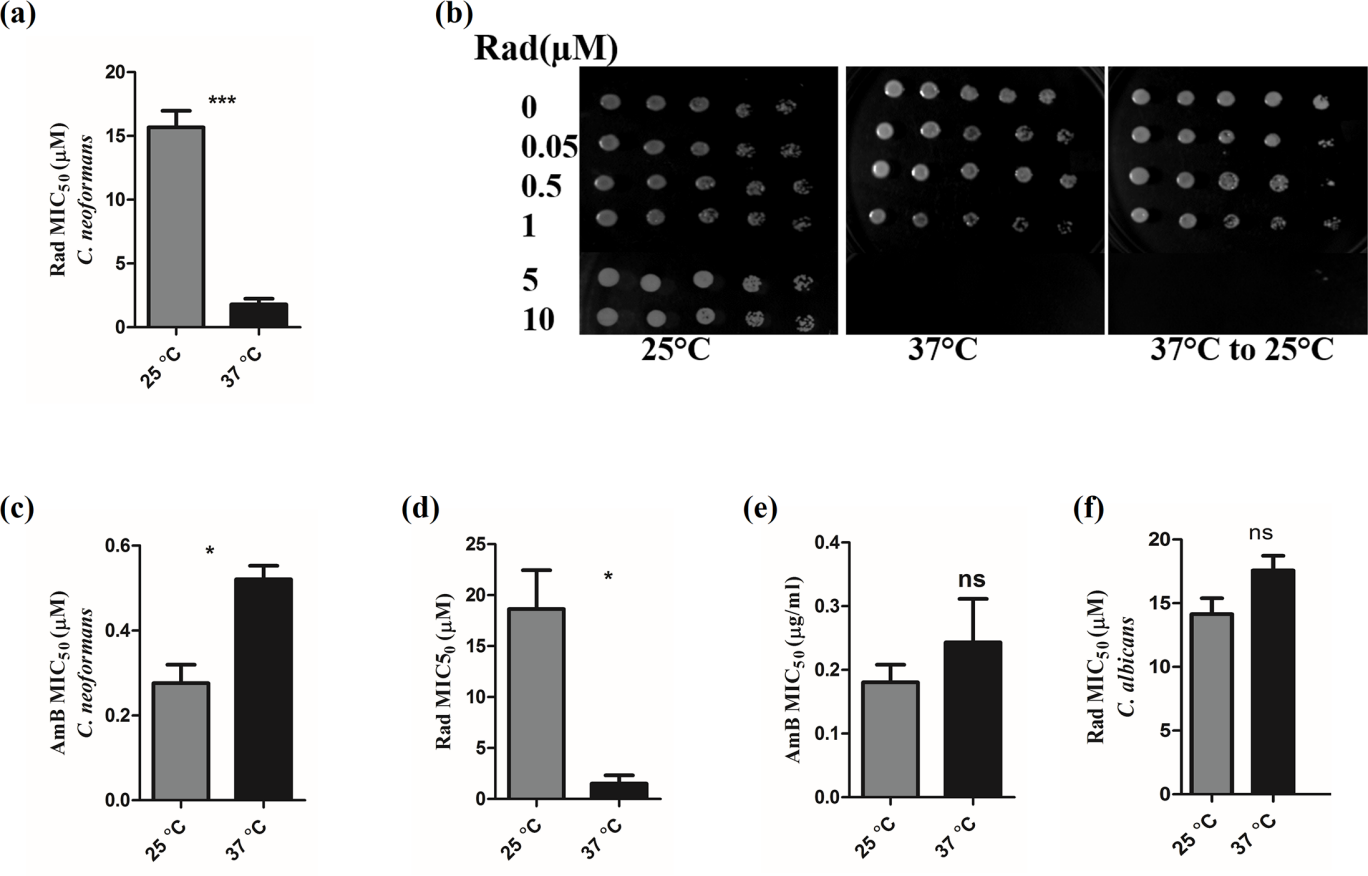
Hsp90 governs thermotolerance in *C*. *neoformans*. (a) Cells were grown in the presence of Hsp90 inhibitor RAD at various concentrations ranging from 5nM to 100 μM at the temperatures indicated above. Comparison of MIC_50_ values for RAD at 25°C and 37°C clearly indicates that *C*. *neoformans* growth is hypersensitive to Hsp90 inhibition at 37°C. (b) Spot agar assay without inhibitors indicate that Hsp90 inhibition is fungicidal at 37°C. Growth is not seen when cells grown in presence of RAD at 37°C were spotted and incubated at 25°C and 37°C. (c) Comparison of MIC_50_ values for antifungal drug Amphotericin B shows similar susceptibilities at both temperatures ruling out lower MIC value due to enhanced drug uptake at higher temperature. (d) *C*. *neoformans* clinical isolate shows 12.4-fold hypersensitivity to Hsp90 inhibition at 37°C. (e) *C*. *neoformans* clinical isolate show similar susceptibility to AmB at both temperatures tested ruling out nonspecific effect. (f) Comparison of MIC_50_ values for RAD in *Candida albicans* at 25°C and 37°C clearly indicates that *C*. *albicans* growth is not hypersensitive to Hsp90 inhibition at 37°C.

The first and foremost criterion for a pathogen to establish infection in any host is its ability to survive and propagate successfully at the physiological temperature of the host. To probe the role of Hsp90 in regulating growth at different temperatures, we tested the effect of Hsp90 inhibitor Radicicol (RAD) on the growth of *C*. *neoformans* at both 25°C and 37°C. MIC_50_ (minimum inhibitory concentration) for RAD treatment was observed to be 15.67 μM when cells were grown at 25°C. However, at 37°C, there was almost a tenfold reduction in MIC_50_ (Fig 1A). At 37°C, MIC_50_ was found to be 1.78 μM. This indicates that *C*. *neoformans* critically depends on Hsp90 for survival at 37°C as the dose which causes no inhibition at 25°C is more potent at 37°C. To test whether compromise of Hsp90 function can exert a fungistatic or fungicidal effect at 37°C, RAD susceptibility testing was followed by spotting on agar plates without any inhibitor. Cells grown at 37°C for 72 hours in presence of RAD were spotted and incubated both at 25°C and 37°C. We found that *C*. *neoformans* grown at 37°C for 72 hr in the presence of RAD were inviable after transferring to 37°C as well as 25°C indicating that the Hsp90 inhibition is fungicidal (Fig 1B). To rule out enhanced drug uptake at higher temperature, we determined the effect of Amphotericin B (AmB) on growth at 25°C and 37°C (Fig 1C). MIC_50_ values for AmB was 0.27μg/ml and 0.52 μg/ml at 25°C and 37°C respectively indicating that hypersensitivity to Hsp90 inhibitor observed at 37°C is not a general phenomenon.

A more pronounced effect was seen for a *Cryptococcus* clinical isolate wherein MIC _50_ value was found to be 18.63 μM and 1.51 μM at 25°C and 37°C respectively (Fig 1D). AmB MIC_50_ values remained the same at both temperatures (Fig 1E). At higher concentrations of drug, there was no growth at both temperatures tested, indicating Hsp90 function is essential in *C*. *neoformans*. Next, we wanted to address whether high temperature hypersensitivity is specific to *C*. *neoformans*. We did the same growth inhibition assay in presence of RAD with *C*. *albicans* as an organismal control wherein we found that MIC values remain the same at both temperatures tested (Fig 1F). We speculate that the chaperone machinery which includes cochaperones and clients may be different in thermotolerant and non-thermotolerant *Cryptococcus* species. Thus, the increased sensitivity of *C*. *neoformans* to Hsp90 inhibition at physiological temperature clearly suggests that it is critically dependent on the Hsp90 machinery for thermotolerance in its host which is the first step for infection.

### CnHsp90 is a functional ATPase

**Fig 2:**
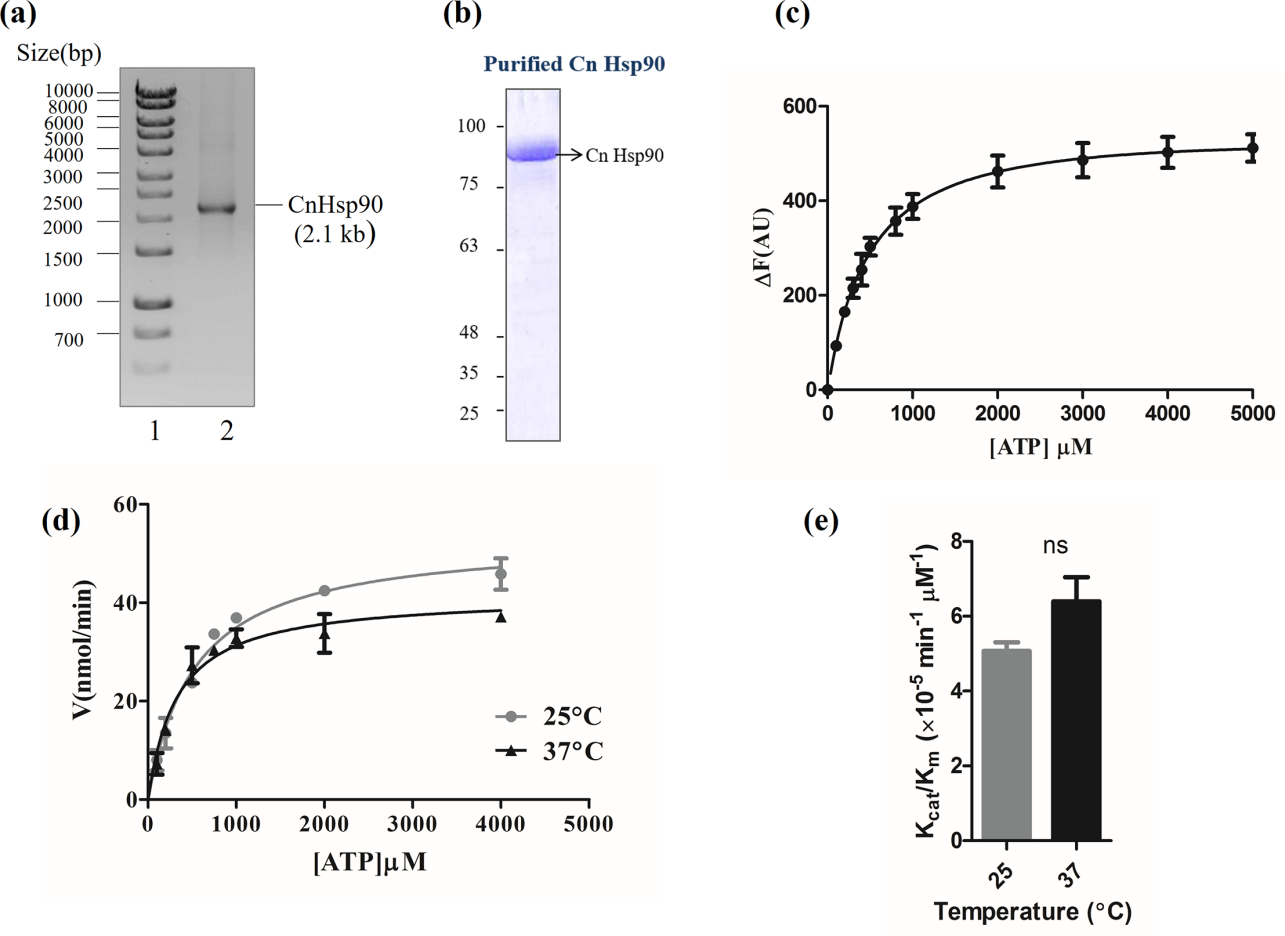
CnHsp90 is a functional ATPase. (a) Cloning of *Cryptococcus* Hsp90 in pRSET-A vector. Lane1, DNA ladder; lane 2, insert (2.1 kb CnHsp90). (b) Coomassie-stained gel showing purified fraction of recombinant His-tagged CnHsp90 protein obtained using Ni-NTA chromatography (molecular mass, ~86 kDa). (c) Binding affinity of cognate ligand ATP to CnHsp90 using tryptophan fluorescence quenching assay. Change in intrinsic fluorescence intensity upon ligand binding was plotted against ligand concentration. Dissociation constant, K_d_, for ATP binding was found to be 497.05 μM. (d) Rate of ATP hydrolysis at 25°C and 37°C was measured by monitoring the hydrolysis of radiolabelled ATP to ADP. A Michaelis–Menten plot shows the fractional cleavage of γ-32P-labeled ATP plotted against ATP concentration. (e) Catalytic efficiency of CnHsp90, Kcat/Km, was found to be 5.07 × 10^−5^ min^−1^ μM^−1^ at 25°C and 6.39 × 10^−5^ min^−1^ μM^−1^ at 25°C and 37°C, respectively. Thus, the ATPase activity is slightly higher at 37°C.

To further delineate the role of Hsp90 in governing thermotolerance, we investigated whether temperature can enhance the function of *C*. *neoformans* Hsp90 (CnHsp90) at the biochemical level. Hsp90 function depends on its ATPase activity and N-terminus has a unique Bergerat-type ATP-binding fold that is involved in ATP binding. A critical arginine residue positioned in the middle domain of the protein occupies the centre stage in catalysis [28]. At the level of primary structure, *C*. *neoformans* Hsp90 shows 69% and 68% identity with *C*. *albicans* and human Hsp90 respectively. However, it is a well-established fact that Hsp90s from different organisms differ in their biochemical activities. Furthermore, it has been previously shown that protozoan parasites have higher ATPase activity than their human counterpart. [3,6,24,29]. The biochemical parameters of Hsp90 from fungal pathogens have not been characterized previously. Therefore, to determine the biochemical properties of CnHsp90, it was cloned from *C*. *neoformans* genomic DNA in pRSET-A vector (Fig 2A). CnHsp90- 6×-His tagged fusion protein was expressed in *Escherichia coli* BL21 pLysS and was purified to homogeneity using Ni-NTA chromatography (Fig 2B).

Intrinsic fluorescence quenching was employed to analyse CnHsp90 binding to its ligand ATP. Briefly, increasing concentration of ATP was incubated with a constant amount of protein and the decrease in the intrinsic fluorescence of protein upon binding to ligand was monitored as described under “experimental procedures”. Difference in intrinsic fluorescence was plotted against molar concentration of the ligand and the saturation curve thus obtained was further analysed by Graphpad Prism 5 software using non-linear regression analysis. CnHsp90 was found to display measurable differences in fluorescence intensity induced by varying concentrations of ATP (Fig 2C). The dissociation constant (K_d_) for ATP binding was determined to be 497.05 μM.

Next, we wanted to determine the ATPase activity of CnHsp90 at 25°C and 37°C. Hydrolysis reaction was performed by incubating CnHsp90 with ATP (100–4000 μM) at 25°C and 37°C. γ-32P-ATP was used as a tracer. Fractional cleavage of ATP by CnHsp90 was plotted against corresponding ATP concentrations in order to calculate the ATPase activity. CnHsp90 obeys Michaelis Menten kinetics and K_m_ for ATP hydrolysis for CnHsp90 were found to be 324.95 μM and 526.85 μM respectively at 25°C and 37°C (Fig 2D). The catalytic efficiency of CnHsp90 was found to be 5.07 × 10^−5^ min^−1^ μM^−1^ at 25°C and 6.39 × 10^−5^ min^−1^ μM^−1^ at 37°C (Fig 2E). The catalytic efficiency of CnHsp90 was found to be higher than human Hsp90. Higher *in vitro* catalytic efficiency indicates that the rate of client cycling through the chaperone cycle will be higher, however, *in vivo* it may also be regulated by cochaperones and cofactors.

### 17-AAG inhibits ATPase activity of CnHsp90 more potently at 37°C

**Fig 3:**
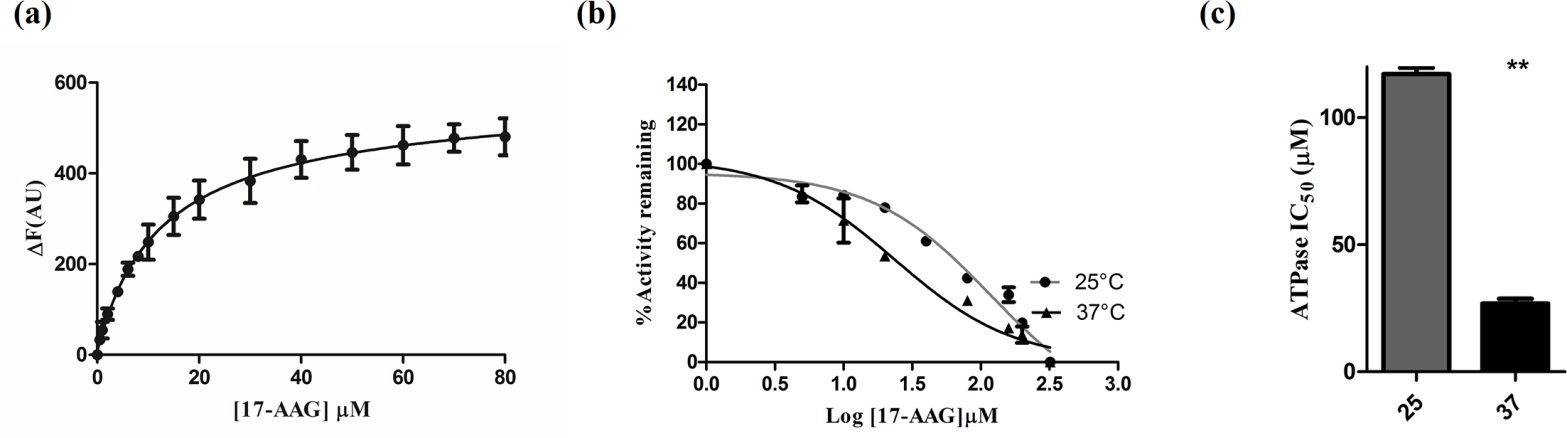
Binding of Hsp90 inhibitor 17-AAG and its effect on CnHsp90 ATPase activity. (a) Binding affinity of competitive inhibitor 17-AAG to CnHsp90 using tryptophan fluorescence was determined. Change in intrinsic fluorescence intensity upon ligand binding was plotted against ligand concentration. Dissociation constant, K_d_, for 17-AAG binding was calculated to be 12.92 μM. (b) IC_50_ for inhibition of ATPase activity of CnHsp90 was determined by incubating the pure protein with fixed, saturating concentration of ATP and varying concentrations of 17-AAG. The reaction was carried out at 25°C and 37°C. The percent activity remaining was plotted against concentration of 17-AAG in logarithmic scale to obtain the inhibition curve. 17-AAG mediates inhibition of CnHsp90 activity at both temperatures tested. (c) IC_50_ values obtained at 25°C and 37°C for CnHsp90 inhibition by 17-AAG was found to be 26.89 μM and 117.15 μM respectively. Therefore, 17-AAG inhibits CnHsp90 more potently at 37°C.

Inhibition of Hsp90 ATPase activity compromises Hsp90 function and hence cell growth. So, we wanted to address whether the observed hypersensitivity to Hsp90 inhibitor at the physiological temperature is also reflected at the level of protein activity. First, we characterized the *in vitro* binding of *C*. *neoformans* Hsp90 to 17-*N*-allylamino-17-demethoxygeldanamycin (17-AAG) which is a derivative of Geldanamycin. 17-AAG and geldanamycin both binds to the nucleotide binding pocket and competitively inhibit the ATPase activity. Geldanamycin is hepatotoxic for clinical use and 17-AAG is known to be a better tolerated derivative. We took the approach of intrinsic fluorescence quenching as described above to determine the binding strength of 17-AAG to Hsp90 *in vitro*. Significant quenching of intrinsic fluorescence of CnHsp90 was seen upon increasing concentrations of the inhibitor. The saturation curve of CnHsp90-17-AAG binding was used to calculate the dissociation constant, K_d_, for 17-AAG binding, which was found to be 12.92 μM (Fig 3A).

Further we determined the effect of 17-AAG on the ATPase activity of CnHsp90. Pure protein was incubated at both temperatures with fixed saturating concentrations of ATP, and 17-AAG concentration was varied, ranging from 5 μM to 300 μM. Percent remaining activity for each 17-AAG concentration was calculated and was plotted against log_10_ 17-AAG concentration (Fig 3B). IC_50_ for 17-AAG-mediated inhibition of CnHsp90 ATPase activity was observed to be 117.15 μM and 26.89 μM at 25°C and 37°C respectively (Fig 3C). Thus at 37°C, 17-AAG inhibits CnHsp90 protein more potently. However, since *in vivo* ATPase activity of Hsp90 is known to be regulated by many cochaperones which either stimulate or bring down Hsp90 ATPase activity, the difference in ATPase IC_50_ observed *in vitro* is not sufficient alone to explain the observation at the cellular level. For example, Sba1p is a cochaperone which stabilizes the ATP bound conformation of Hsp90 by interfering with ATP hydrolysis, thereby prolonging the association of clients with Hsp90 [30]. Aha1 has a ATPase stimulatory activity [31]. Our study is the first to report the kinetic parameters of Hsp90 from a pathogenic fungus. The biochemical properties of CnHsp90 have been tabulated and compared to Hsp90 from host human and protozoan parasites in Table 1.

**Table 1:** Comparison of biochemical properties of Hsp90 from *C*. *neoformans* and comparison with Hsp90 from other organisms [3,6,24,29].

Organism	ATP			17-AAG	
	Km	kcat/Km	K_d_	K_d_	IC_50_
	(μM)	(min^-1^μM^-1^)	(μM)	(μM)	(μM)
*H*. *sapiens*	324	4.6 ×10^−5^	240	4.4 *	0.702*
*C*. *neoformans*	526.85	6.39 x 10^−5^	497.05	12.92	26.89
*P*. *falciparum*	611	16.2 ×10^−5^	168	1.05 *	0.207*
*G*. *lamblia*	894	4.4 ×10−^5^	626	17.06	-
*E*. *histolytica*	246	4.35 ×10^−4^	365.2	10.77	30.9**
*T*. *annulata*	-	2.79x10^-5^	178.6	-	20.59 **
*T*. *evansi*	-	9.3x10^-5^	-	-	-

*GA

**AAG

### Hsp90 is localised to the surface of *C*. *neoformans*

**Fig 4:**
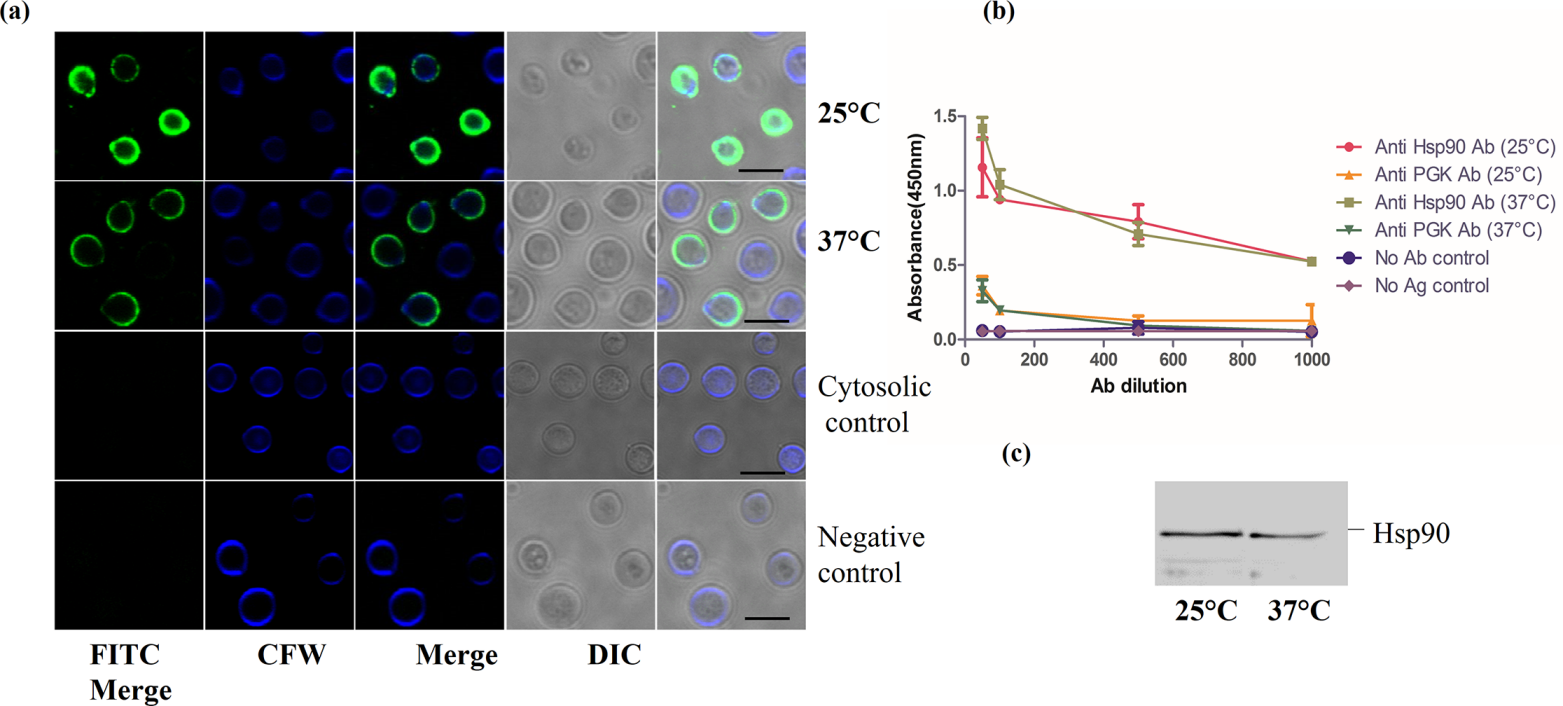
Hsp90 is associated with the fungal cell wall. (a) Exponential phase cells were grown at 25°C and 37°C and subjected to indirect immunofluorescence without permeabilization using specific antibody raised against CnHsp90. Hsp90 was found to be localized at the cell surface. Colocalization of CFW (chitin staining) and FITC signal (Hsp90 staining) is also observed indicating cell wall localization of CnHsp90. Antibody to cytosolic protein PGK and pre-immune sera served as negative controls for the experiments. b) Association of Hsp90 at the cell surface was also probed by whole cell based ELISA wherein plates were incubated with intact yeast cells. Significant binding of CnHsp90 Ab to cell surface Hsp90 was seen at both 25°C and 37°C. Anti PGK Ab was used as a negative control. c) Immunoblot analysis of *C*. *neoformans* βME cell surface extract also detected a band corresponding to Hsp90 further confirming the presence of Hsp90 in the cell wall fraction.

Presence of a melanised cell wall and polysaccharide capsule are the other two well established pathogenicity armours of *Cryptococcus* [32]. Armoured intricately around the cell wall, capsule defends phagocytic action of macrophages [33–35]. While Hsp90 is primarily a cytosolic chaperone, in many pathogenic organisms, it has been shown to be present on the cell surface as well. Most notable among these examples and closely related to our system is *C*. *albicans* wherein a 47kDa, C terminal fragment of Hsp90 was detected on the cell surface [13]. Interestingly, the surface exposed Hsp90 could also be targeted by a monoclonal antibody called Mycograb which was shown to neutralize *Candida* infections [15]. Also, cell surface Hsp90 has been detected in cancer cells [36]. With this background, we therefore examined whether Hsp90 is localised at the cell surface. *Cryptococcal* cells grown at 25°C and 37°C were subjected to indirect immunofluorescence analysis without permeabilization and the association of Hsp90 at the cell surface was probed using specific antibody against CnHsp90, anti CnHsp90 Ab. Hsp90 was observed to be localised on the fungal cell surface at both 25°C and 37°C. Also, a high degree of co-localization between Hsp90 and calcofluor staining was observed implicating that Hsp90 is also enriched in the cell wall (Fig 4A). In order to rule out nonspecific binding of antibody to the fungal cell surface and as a negative control to rule out accidental permeabilization during the experimental steps, we used antibody to a cytosolic protein phosphoglycerate kinase. Immunofluorescence using Anti-PGK Ab was performed as above and signal corresponding to cell surface was not observed in this case. Signal at the cell wall was also not seen in case of cells probed with pre-immune serum thereby ruling out nonspecific antibody binding.

To further reconfirm our results, we examined the surface distribution of Hsp90 by whole cell based ELISA. In brief, polystyrene plates were incubated with intact yeast cells and the cell surface association of Hsp90 was probed with different dilutions of Anti CnHsp90 Ab. As a control to rule out nonspecific binding due to cell lysis, we used an antibody to PGK as above. We saw significant binding of cell surface Hsp90 to the specific antibody as can be seen in the ELISA curve (Fig 4B). Binding was not observed in control fungal cells using polyclonal antiserum against PGK.

To further probe into the nature of this association, we resorted to previously described method of fungal cell “shaving”. β-mercaptoethanol extraction of cell surface associated proteins were performed as described in the experimental procedures. Briefly log phase cells were incubated in 1% βME- PBS for 60 mins and the extract thus obtained (culture supernatant) was subjected to immunoblot analysis using Anti CnHsp90 Ab. A band at the correct molecular size of Hsp90 was detected in the βME extract at both temperatures, further strengthening our observation (Fig 4C).

### Cell surface association of Hsp90 depends on ER Golgi secretory pathway

Capsule and cell wall are intricately linked and assembly of capsule involves the trafficking of a large number of polysaccharides along with the proteins necessary for assembly onto the cell surface. Role of classical secretion pathway has been implicated in capsular monomer assembly by using conditionally defective secretory mutants wherein cargo from accumulated vesicles was found to react with anti GXM mAbs [37]. Interestingly, it was also shown that colloidal gold labelled mAbs against GXM binds with linear fibres present both on cell surface and cytosolic secretory vesicles [38].

**Fig 5:**
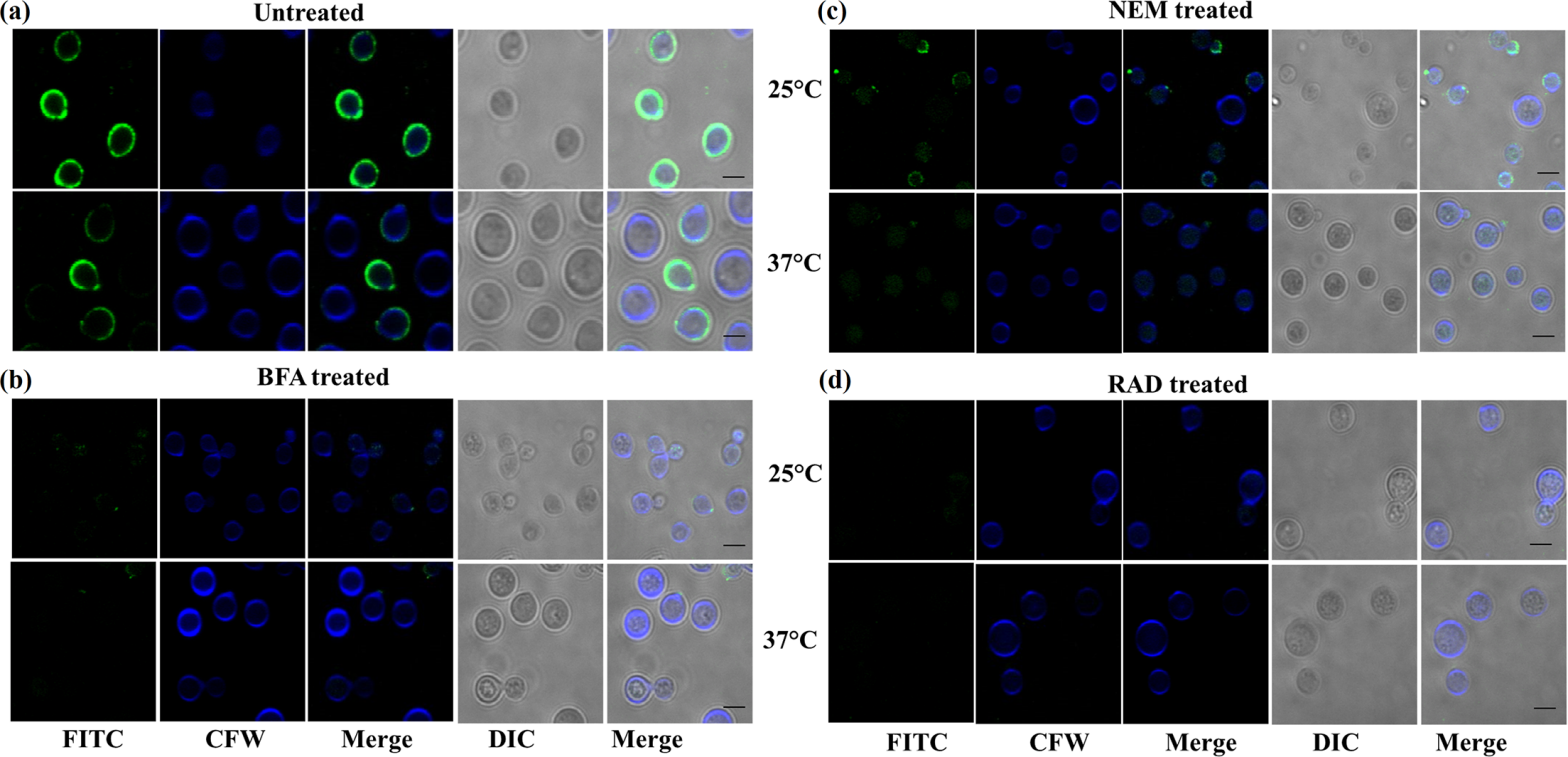
Cell surface association of Hsp90 depends on ER Golgi classical secretory pathway. Indirect immunofluorescence was carried out using Anti CnHsp90 Ab after treatment with different inhibitors. In each case first panel corresponds to cells grown at 25°C and second panel represents cells grown at 37°C. (a) Indirect Immunofluorescence using specific antibody show surface localization of Hsp90 at both temperatures. (b) Cells were treated with vesicle trafficking inhibitor BFA and immunoflourescence was carried out as above. Complete loss of surface signal for Hsp90 was seen in the treated cells both at 25°C (first panel) and 37°C (second panel). (c) Disulfide blocker NEM also abrogated Hsp90 localisation at cell surface. At 25°C diffused signal is seen whereas at 37°C there is no signal thereby implicating the involvement of ER Golgi classical secretion pathway in the process. (d) Hsp90 may be involved in chaperoning a client protein which gets localised to the cell wall. To test this hypothesis, RAD was used to abrogate client association with Hsp90. Loss of cell surface localization was seen in case of RAD treated cells indicating pharmacological inhibitor can also interfere with the process of Hsp90 transport to cell surface.

In order to address how Hsp90 is trafficked to the cell surface, we tried to interfere with the process of capsular assembly by using Brefeldin A (BFA), a well-studied vesicle trafficking inhibitor. BFA interferes with the action of ARF and blocks the anterograde transport of proteins between the ER and Golgi apparatus. Cells were grown in presence of BFA at 25°C and 37°C and then immunofluorescence was carried out as described above. Signal for Hsp90 at cell surface was completely abolished in BFA treated cells (Fig 5A and 5B) indicating ER to Golgi transport is important for Hsp90 localization to the cell surface. We also used N-ethylmaleimide (NEM) which is a cysteine alkylating agent that interferes with disulphide bond formation. A faint and diffused signal is seen in case of NEM treated cells which indicates NEM is also able to abolish the cell surface trafficking process of Hsp90 (Fig 5C). This result clearly establishes that cell surface association of Hsp90 depends on the classical secretory pathway.

We hypothesized that Hsp90 may be chaperoning client proteins which are important in this process and hence gets piggybacked along with its client to the surface. This means Hsp90 inhibition will abrogate its association with these client proteins and hence would interfere with the process of its trafficking to the cell wall. To test this possibility, we also grew the cells in the presence of 0.5 μMRAD for 48 hours and probed Hsp90 surface localisation as above. We examined the cell viability by Trypan blue dye exclusion method and found 90–95% viability thus ruling out cell death during RAD treatment. Signal at the cell surface for Hsp90 was not observed at both temperatures (Fig 5D). This indicates that pharmacological inhibition of Hsp90 also leads to derailment of the pathways necessary for its localization on the cell wall.

### Hsp90 regulates capsule induction and maintenance in *C*. *neoformans*

**Fig 6:**
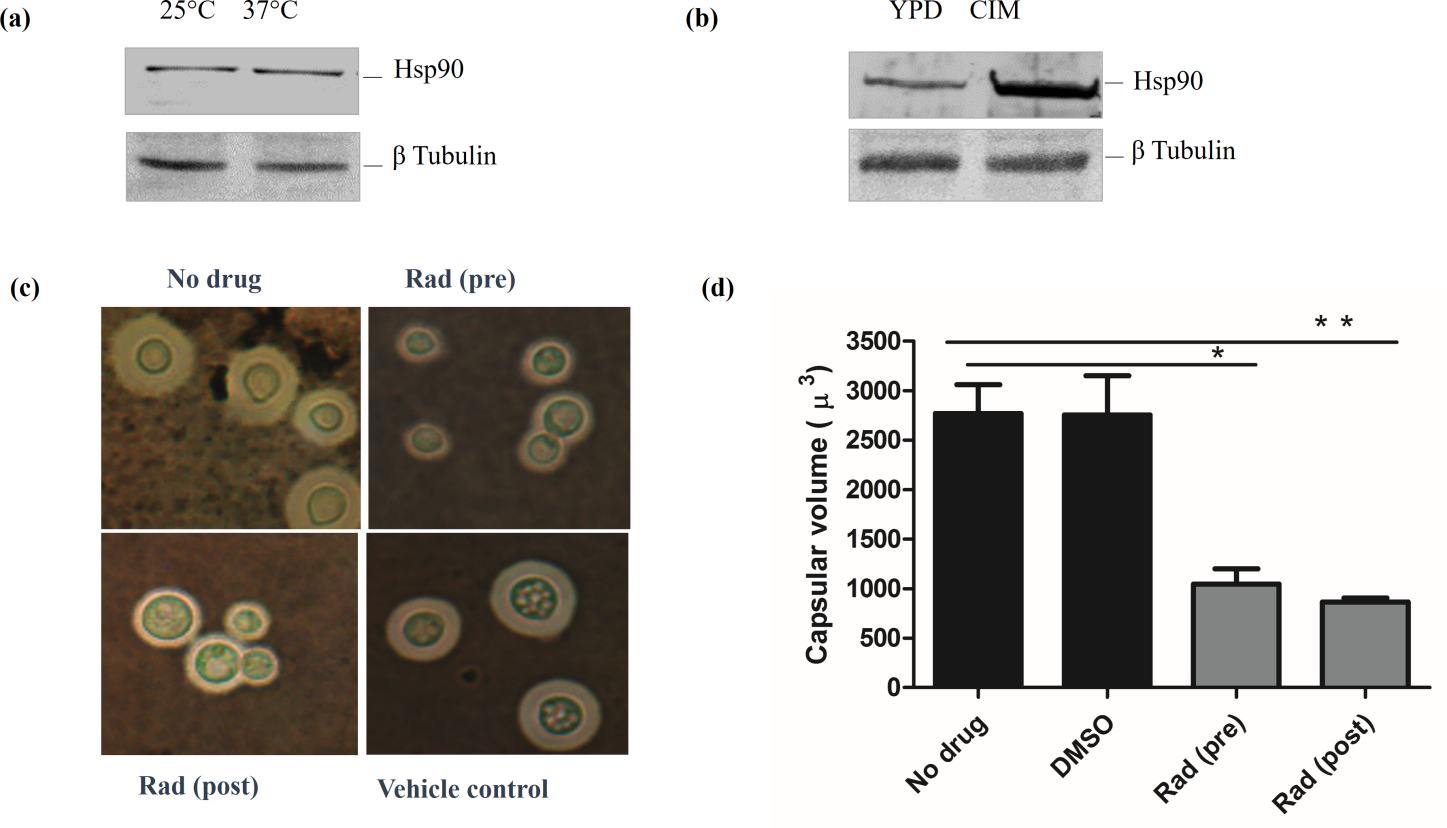
Hsp90 is involved in induction and maintenance of capsule in *C*. *neoformans*. (a)Western blot analysis to probe levels of Hsp90 at 25°C and 37°C indicate similar expression levels at both temperatures tested. (b) Hsp90 is significantly upregulated under capsule inducing conditions. (c) Representative microscopic images showing compromise of Hsp90 function during or after capsule induction leads to drastic compromise in capsular size. (d) Hsp90 is involved in capsule assembly as well as maintenance around the cell wall as pre-and post-treatment with RAD leads to approximately 60% decrease in capsular volume relative to no drug and vehicle control.

Cells respond to stress by regulating the abundance of proteins in myriad ways. Therefore, to get a cellular picture of Hsp90 under thermal stress, we first quantified the protein level expression of Hsp90 at different temperatures. Cells were grown at 25°C and 37°C till exponential phase and the lysate was probed with Anti CnHsp90 antibody. Upon checking the expression of CnHsp90 under steady state, we found similar protein levels under conditions of prolonged growth in YPD at 25°C and 37°C (Fig 6A). When cells were grown at 25°C and then shifted to 37°C, we found transient increase in Hsp90 levels after 20 mins (S1A Fig). However, since *in vitro* culture conditions are drastically different than the host environment, we tried to mimic the host environment by growing cells in the presence of 10% serum, which also is known to induce the process of capsulation. We probed protein levels under capsule inducing conditions and found a marked upregulation of Hsp90 under these conditions (Fig 6B). We also observed significant upregulation of Hsp90 under another well-established capsule inducing condition i.e. 37°C and 5% CO_2_ which mimics lung environment (S1B Fig).

Capsule regulation is dynamic, it’s size changes in context of infection and polysaccharide shedding is also reported. Since Hsp90 is cell surfaced localised and is also upregulated under capsule inducing conditions, therefore we hypothesized that compromise in Hsp90 function may affect capsular assembly. To test this hypothesis, we examined the effect of Hsp90 inhibition on capsule formation. First cells were incubated in capsule inducing medium in presence of RAD and capsule formation was observed microscopically with respect to no drug control. Significant reduction in capsule size was observed when RAD was added in the capsule inducing media as compared to no drug (Fig 6C). This indicates that Hsp90 function is important for induction of capsule formation as treatment with RAD during capsule induction led to approximately 60% reduction in capsular volume (Fig 6D). Next, we asked if Hsp90 is also required for maintenance of capsule around the cell. Cells were first grown in capsule inducing medium and RAD treatment was done post capsulation. Similar reduction was seen in capsular volume in case of post capsulation treatment also indicating that Hsp90 is required for maintenance of capsules around the cell wall. Taken together, these observations clearly suggest that Hsp90 trafficking to the cell wall and capsular assembly are related processes and hence it assigns an important role of regulating capsule dynamics to Hsp90 in *C*. *neoformans*.

### Hsp90 governs echinocandin resistance at 37°C but not at 25°C

**Fig 7:**
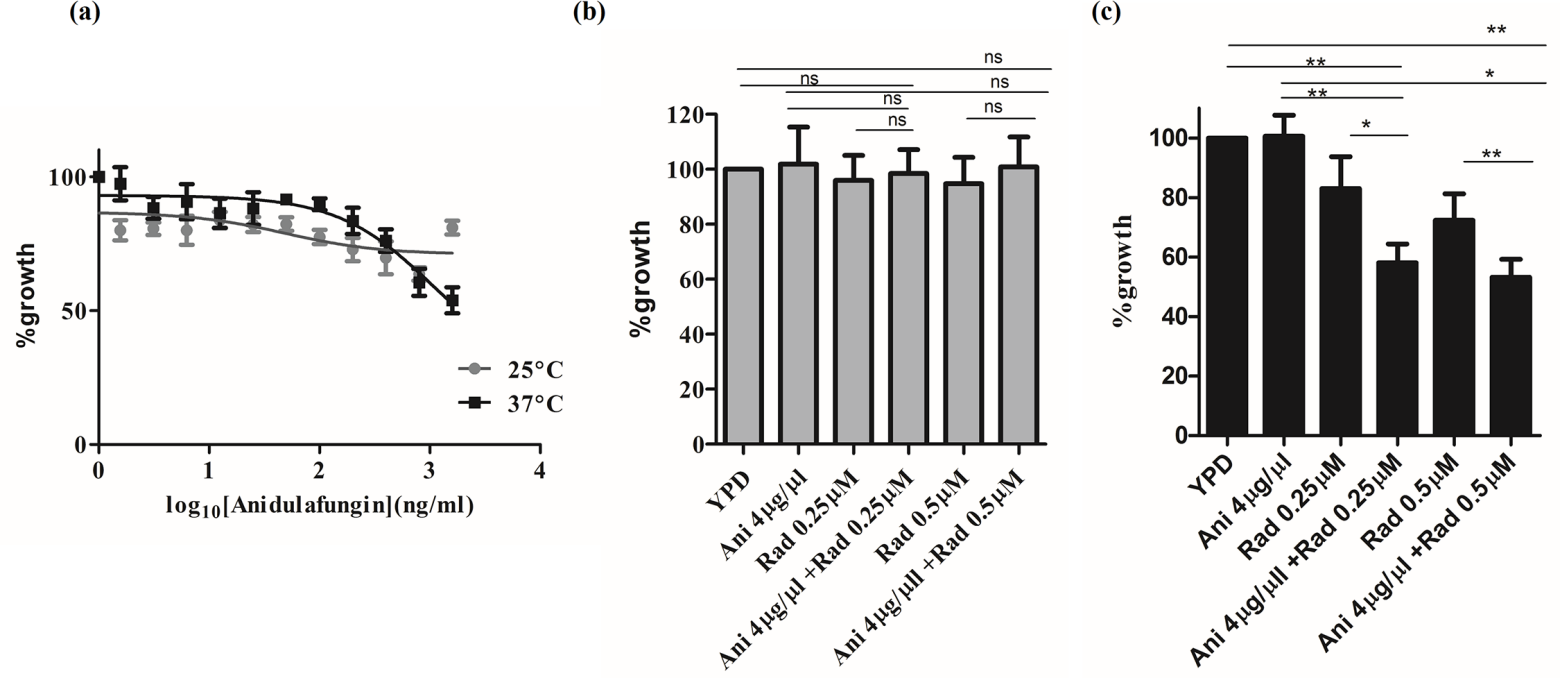
Hsp90 plays a crucial role in intrinsic anidulafungin tolerance of *Cryptococcus neoformans*. (a) *In vitro* antifungal susceptibility of *C*. *neoformans* to anidulafungin was evaluated at both 25°C and 37°C. Growth in presence of anidulafungin was seen even at high concentrations of the drug indicating robust intrinsic resistance. (b) MIC assay was performed with AF and Hsp90 inhibitor RAD at the concentrations indicated and incubated for 72 hours at 25°C. Growth was not found to be sensitive to combination of both drugs at 25°C. (c) Pharmacological inhibition of Hsp90 with RAD at 37°C reduces tolerance to AF at 37°C indicating additive interaction between these two drugs.

*Cryptococcus* has been shown to be intrinsically resistant to echinocandins which target the fungal cell wall. In *Candida albicans* and *Aspergillus fumigatus*, role of Hsp90 in echinocandin resistance has been well established [10]. Previous studies in *Cryptococcus* have shown synergy of Hsp90 inhibitors Mycograb [15] and RAD [18] with azoles. Given that Hsp90 is present at the surface of *C*. *neoformans*, we set out to examine if Hsp90 has a role in potentiating echinocandin resistance in *Cryptococcus*. To address this, first we evaluated the susceptibility of *C*. *neoformans* to the widely used echinocandin–anidulafungin(AF) by performing antifungal broth microdilution assays as described previously. This assay was done both at 25°C and 37°C to examine whether temperature influences AF tolerance. We observed robust resistance to AF as indicated by normal growth of cells even at high doses of AF (16μg/ml). Also, higher temperature had no impact on AF tolerance as resistance was seen at both temperatures tested (Fig 7A). Therefore, *C*. *neoformans* is resistant to AF and the resistance profile is similar at 25°C and 37°C.

Next, we wanted to evaluate the impact of pharmacological inhibition of Hsp90 on AF resistance in *Cryptococcus*. A similar approach was followed wherein cells were grown in presence of AF and RAD at the concentrations indicated (Fig 7B). Compromising Hsp90 function with RAD (0.25μM and 0.5 μM) in presence of AF (4μg/ml)) had no impact on growth of the isolate as compared to the drug free controls at 25°C. Notably, at 37°C there was approximately a 42% decrease in growth at a lower dose of AF (4μg/ml) upon RAD treatment at a concentration of 0.25μM. At 0.5μM RAD, AF had enhanced sensitivity with almost 47% lower growth (Fig 7C). Surprisingly this effect was not seen at 25°C at the same concentrations tested as mentioned above. To determine the nature of drug interaction, we further analyzed the fractional inhibitory concentrations of the two drugs at 37°C by calculating ∑FIC for the combination treatment as described in the methods section. ∑FIC value for RAD and AF combination was calculated to be 0.56 thus indicating an additive effect. This strengthens the fact that Hsp90 mediated thermotolerance regulates pathogenicity of the fungus in myriad ways. Our study therefore also reinforces the potential of Hsp90 inhibitors as a promising and powerful antifungal combination therapy along with echinocandins against *Cryptococcal* infections.

## Discussion

Fungal pathogens first need to cross the formidable barrier of high physiological temperature in order to live and colonize within mammals. One such example of thermotolerance manoeuvring fungal pathogenesis has been seen in the genus *Cryptococcus* wherein only species capable of growth at 37°C are human pathogens. *Cryptococcus* species which lack this ability despite having other virulence factors, for example, *C*. *podzolicus* are non-pathogenic [39]. Temperature is known to act as a cue for dimorphism in *C*. *albicans* wherein it undergoes yeast to hyphal transition at 37°C which is implicated in its virulence. Interestingly Hsp90 has been shown to be the regulator of this switch by repressing the Ras1-PKA signaling pathway [7]. In *C*. *neoformans*, serial analysis of gene expression and microarray studies have revealed the upregulation of Hsp90 at 37°C [22,40], under reduced iron conditions [41] and under fluconazole stress [42]. However detailed understanding about the role of Hsp90 in *C*. *neoformans* is lacking. In this context, we have investigated the role of Hsp90 in governing thermotolerance in *C*. *neoformans*. We find that growth at physiological temperature critically depends on Hsp90 function as mild compromise in function at 37°C but not at 25°C is lethal for *C*. *neoformans*. We further investigated biochemical profile of the protein by characterization of its ATPase activity and inhibition by 17-AAG. This is the first report of the kinetic parameters of Hsp90 of a pathogenic fungus. We found that CnHsp90 binds to its ligand with an affinity constant 497.05 μM. ATPase activity was found to be higher at 37°C. This strengthens the fact that being a pathogen which experiences stressful and highly demanding environment, higher rate of client cycling through the chaperone cycle is a must as reflected by higher catalytic efficiency. Interestingly we observe that ATPase activity is 4.5-fold more sensitive to inhibition by 17-AAG at 37°C which indicates different conformational dynamics of the protein at higher temperature. Nonetheless, this agrees with higher cellular growth inhibition indicating at 37°C, ATPase activity of Hsp90 is more crucial for its survival.

Alterations in ambient temperature may lead to a change in concentration of free Hsp90 as global burden of protein folding increases and hence may influence the interaction of Hsp90 with those clients which are required for growth at high temperature. This prompted us to investigate the localization of Hsp90. Hsp90 was found to be cell surface associated and this association was perturbed by interfering with the ER Golgi classical secretory pathway. Since cell wall is also intricately associated with capsule, another important and unique virulence attribute of the pathogen, we probed Hsp90 levels under capsule inducing conditions and found it to be upregulated. Capsular assembly is a complicated process as it involves synthesis of nucleotide sugar donors in the cytoplasm, however, assembly takes place near the cell wall. Active transport of capsular polymers takes place and it has been shown that inhibitors of vesicular transport or secretory pathway mutants lead to decreased capsule. Additionally, the cell wall is the interface of capsule attachment and it provides a scaffold for proteins that mediate the process. Upon compromise of Hsp90 function during the process of capsulation as well as post capsulation, significant reduction in capsule size was seen thereby confirming that Hsp90 is involved both in assembly and maintenance of capsule. In this light, presence of Hsp90 on the cell wall indicates that it chaperones important clients which are involved in the above processes, hence, compromising Hsp90 function compromises cell wall organisation which in turn affects capsule assembly. Pharmacological inhibition of Hsp90 may titrate it away from the client which gets degraded and hence we see a decrease in capsule formation. Signalling pathways mediated by kinases like Hog1, PKC and protein kinase A have been shown to be involved in capsule regulation in *Cryptococcus*. These kinases are well established clients of Hsp90 in other organisms such as *C*. *albicans*. We speculate that under capsule inducing conditions, Hsp90 chaperones the dephosphorylated form of Hog1 and keeps it poised for signal transduction to regulate capsule size. In yeast, a dedicated cochaperone called Cdc37 which presents kinase clients to Hsp90 was found to be upregulated at the transcript level in capsular mutants under capsule inducing conditions. It would be interesting to experimentally validate the interaction of Hsp90 and Cdc37 with these kinases in *Cryptococcus*. Transcript data analysis (available in FungiDB) also revealed upregulation of another co-chaperone Aha1. Nonetheless, different line of evidences such as cell surface localisation of Hsp90, upregulation of Hsp90 and cochaperones under different capsule inducing conditions, reduction in capsule volume upon Hsp90 inhibition and transcript data analysis suggests a crucial role of Hsp90 machinery in governing critical aspects of cell wall and capsule regulation in the pathogen.

Echinocandins are the only class of antifungals whose target is located on the fungal cell wall and coincidentally we found Hsp90 to be also localised on the cell wall. Echinocandins are the most recent class of antifungals with a broad inhibitory spectrum against most pathogenic fungi such as *Candida* and *Aspergillus* [43,44]. However, it seems that *Cryptococcus* is intrinsically resistant to Echinocandins. Surprisingly, studies have pointed out that the target of the drug, (1,3) beta-glucan synthase is essential for the organism [45] and also the enzyme is inhibited *in vitro* by echinocandins [46]. Mutations in the target genes FKS1 and FKS2 are also not responsible for the resistant phenotype. Our work reveals Hsp90 inhibitor RAD and anidulafungin are effective in combination against *Cryptococcus* thereby extending the current antifungal armoury. Interestingly this effect is also seen at 37°C and not at 25°C, thus again reconfirming the fact that virulence and thermotolerance are linked in this fungus. This implies that either Hsp90 directly regulates cell wall stress response exerted by echinocandins such that impairing Hsp90 function abrogates intrinsic resistance to echinocandins or Hsp90 is essential for cell wall integrity and inhibition of Hsp90 makes anidulafungin more accessible to its target.

We postulate Hsp90 commands the thermotolerant ability by chaperoning crucial clients directly involved in the process. For example, calcineurin, a bona fide client of Hsp90, has been implicated in growth at high temperature as well as virulence of the pathogen [47–49]. As such, compromising Hsp90 function may cause destabilization of proteins involved in growth at high temperature, which in turn leads to derailment of the pathways enabling thermotolerance. Kinases like Hog1, Mkc1 and Cek1, which are crucial for cell wall integrity [50], are also well- established clients of Hsp90 [12,51]. Taken together, our study establishes a critical role of Hsp90 in mediating three essential virulence determinants in *C*. *neoformans* i.e. thermotolerance, capsule formation and echinocandin resistance. Compromising Hsp90 function may lead to rewiring of fundamental pathways implicated in virulence and hence our study corroborates with numerous other studies highlighting the potential of targeting Hsp90 in fungal pathogens. Understanding the basis of thermotolerance and virulence will thus not only highlight acquisition of pathogenicity determinants, but may also open new therapeutic avenues to cripple a lethal infection.

## Supporting information

S1 Fig Immunoblot showing Hsp90 levels under (a) transient induction conditions at 37°C and (b) 5% CO_2_ capsule inducing condition.
(TIF)
