# Supplementary figures and images for: Heat shock protein 90 localizes to the surface and augments virulence factors of Cryptococcus neoformans
Source: PLoS Negl Trop Dis. 2017 Aug 4;11(8):e0005836. doi: 10.1371/journal.pntd.0005836 (PMC5559104; doi:10.1371/journal.pntd.0005836)

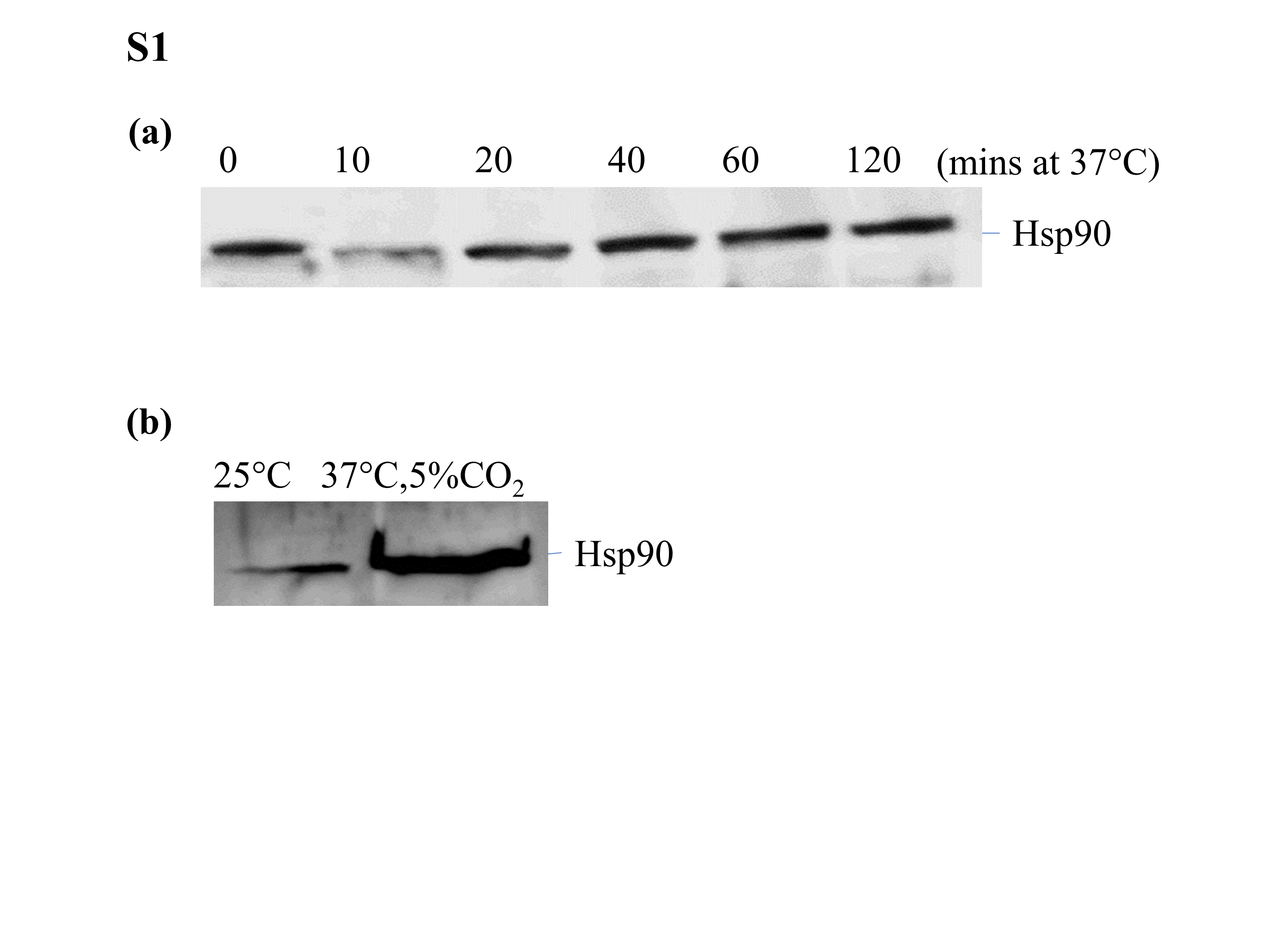

Supplement: S1 Fig — Immunoblot showing Hsp90 levels under (a) transient induction conditions at 37°C and (b) 5% CO2 capsule inducing condition. (TIF) [file pntd.0005836.s001.tif]
